# Serum IL-1β predicts de novo hepatitis B virus reactivation during direct-acting antiviral therapy for hepatitis C, not during anti-cancer/immunosuppressive therapy

**DOI:** 10.1038/s41598-022-21315-z

**Published:** 2022-10-07

**Authors:** Naoki Kawagishi, Goki Suda, Ryotaro Sakamori, Takeshi Matsui, Masahiro Onozawa, Zijian Yang, Sonoe Yoshida, Masatsugu Ohara, Megumi Kimura, Akinori Kubo, Osamu Maehara, Qingjie Fu, Shunichi Hosoda, Yoshimasa Tokuchi, Kazuharu Suzuki, Masato Nakai, Takuya Sho, Kenichi Morikawa, Mitsuteru Natsuizaka, Koji Ogawa, Hajime Sakai, Shunsuke Ohnishi, Masaru Baba, Tetsuo Takehara, Naoya Sakamoto

**Affiliations:** 1 Department of Gastroenterology and Hepatology, Hokkaido University Graduate School of Medicine, Sapporo, Hokkaido Japan; 2 Department of Gastroenterology and Hepatology, Osaka University Graduate School of Medicine, Suita, Osaka Japan; 3 Center for Gastroenterology, Teine Keijinkai Hospital, Sapporo, Hokkaido Japan; 4 Department of Hematology, Hokkaido University Graduate School of Medicine, Sapporo, Hokkaido Japan; 5 Laboratory of Molecular and Cellular Medicine, Faculty of Pharmaceutical Sciences, Hokkaido University, Sapporo, Japan; 6 Department of Gastroenterology and Hepatology, Japan Community Health Care Organization Hokkaido Hospital, Hokkaido, Japan; 7 Department of Hematology, Teine Keijinkai Hospital, Sapporo, Japan

**Keywords:** Cytokines, Biomarkers, Liver diseases, Hepatitis, Viral hepatitis

## Abstract

De novo hepatitis B virus (HBV) reactivation occurs during direct-acting antiviral (DAA) treatment in hepatitis C virus (HCV)-infected patients with resolved HBV infection. We evaluated the predictive factors, mechanical insight, and differences of cytokine levels during anti-cancer/immunosuppressive and DAA. Eleven, 35, and 19 HCV-infected patients with previous HBV infection with HBV reactivation during DAA treatment, previous HBV infection without HBV reactivation during DAA treatment, and without HBV infection resolution receiving DAA treatment, respectively, were enrolled. Clinical data and baseline cytokine levels were analyzed. Low baseline serum interleukin (IL)-1β levels predicted de novo HBV reactivation during DAA treatment (odds ratio: 47.6, 95% confidence interval: 6.94–333.3). HCV-infected patients with the IL-1β gene single nucleotide polymorphism rs16944 AA allele had significantly higher IL-1β levels; no HCV-infected patient with the IL-1β AA allele experienced HBV reactivation during DAA treatment. Compared to HCV-infected patients with HBV infection resolution, non-HCV infected patients with or without HBV reactivation during anti-cancer/immunosuppressive therapy or bone marrow transplantation had remarkably lower baseline IL-1β levels. Low IL-1β levels were not associated with HBV reactivation. IL-1β levels before DAA for HCV-infected patients with resolved HBV infection could predict HBV reactivation during DAA treatment.

## Introduction

The development of direct-acting antivirals (DAAs) for hepatitis C virus (HCV) has dramatically changed the efficacy of anti-HCV therapy compared to the previous standard therapy using interferon (IFN)-based drugs^1,2^. Even in HCV-infected patients who are difficult to treat with IFN-based therapy^3,4^, most could achieve a sustained viral response by DAAs. However, in patients with HBV and HCV co-infection, because DAA does not have anti-hepatitis B virus (HBV) activity, unlike IFN therapy, HBV reactivation has been more occasionally observed^5,6^. Moreover, even in HCV-infected patients with resolved HBV infection, DAA therapy causes HBV reactivation^7^. Importantly, in some cases, HBV reactivation-induced hepatitis could be lethal^6,8^.

HBV reactivation in patients with resolved HBV infection has been well known and analyzed in anti-cancer therapy, immunosuppressive therapy, and organ transplantation^9–11^. HBV reactivation in patients with resolved HBV infection during anti-cancer and immunosuppressive therapies is called de novo HBV reactivation. De novo HBV reactivation causes severe fulminant hepatitis, which is fatal in most cases, at a high rate^9–11^. Thus, rigorous monitoring and treatment of de novo HBV reactivation are required. Similarly, several cases of de novo HBV reactivation during DAA treatment cause severe hepatitis^12–14^.

However, the similarities and differences in mechanisms and risk factors of de novo HBV reactivation between patients receiving immunosuppressive/anti-cancer therapy and HCV-infected patients receiving DAAs remain unclear.

Various cytokines are involved in HBV replication and lifecycle and contribute to HBV clearance^15^. Interleukin (IL)-1β is reported to be involved in reducing HBV covalently close circular DNA (cccDNA)^16^, and IL-6, IL-1β, and tumor growth factor (TGF)-β are involved in suppressing HBV transcriptional activity and replication^15,17,18^. Thus, cytokines and chemokines may be involved in HBV reactivation. However, the association between serum cytokine levels and HBV reactivation in HCV-infected patients with resolved HBV infection during DAA treatment, and the differences between them and patients receiving anti-cancer/immunosuppressive therapy, have not been clarified.

Therefore, we aimed to evaluate the association between serum cytokine levels and HBV reactivation during or after DAA. Additionally, we compared cytokine levels among HCV-infected patients with resolved HBV infection, those with/without HBV reactivation during DAA therapy, and non-HCV-infected patients with resolved HBV infection with/without HBV reactivation during anti-cancer/immunosuppressive therapy.

## Methods

### Patients and study design

#### Cohort 1

In this retrospective study, we screened HCV-infected patients initiated with IFN-free DAAs between April 2014 and May 2020 at Hokkaido University Hospital and Osaka University Hospital. Patients were evaluated for baseline HBV infection parameters, including antibody to hepatitis B core antigen (anti-HBc), hepatitis B surface antigen (HBsAg), anti-HBs, and HBV-DNA levels. Patients with previous HBV infection were defined as negative for both HBsAg and HBV-DNA and positive for anti-HBc and/or anti-HBs without HBV vaccination. This study included three patient groups. First, we included patients with HCV infection who (1) had a resolved HBV infection, (2) were initiated with IFN-free DAA therapy between April 2014 and May 2020, (3) had HBV reactivation or reappearance during DAA therapy, (4) had proper clinical information at baseline and end of treatment, and (5) had preserved serum at baseline. Eleven patients were included in this group.

Second, we included patients with HCV infection who (1) had a resolved HBV infection, (2) were initiated with IFN-free DAA therapy between October 2014 and May 2016, (3) did not have HBV reactivation or reappearance during DAA therapy, (4) had proper clinical information at baseline and end of treatment, and (5) had preserved serum at baseline. From these, we selected 35 patients to serve as controls.

Lastly, we included patients with HCV infection who (1) did not have resolved HBV infection, (2) were initiated with IFN-free DAA therapy between October 2014 and May 2016, (3) had proper clinical information at baseline and end of treatment, (4) had preserved serum at baseline, and (5) had end of treatment (EOT). From these, we selected 19 patients to serve as controls.

Patients were excluded if they were positive for HBV-DNA and/or HBsAg at baseline, received the hepatitis B vaccination, had co-infection with human immunodeficiency virus (HIV), had insufficient clinical information, or did not have preserved serum at baseline.

We defined HBV reactivation as an increase of ≥ 1.3 log IU/mL in serum HBV-DNA levels^9,19^ in patients with resolved HBV infection^5^. HBV reappearance was defined as HBV-DNA detectable, but < 1.3 log IU/mL in patients with resolved HBV infection.

HBV-reactivation hepatitis was defined as HBV-DNA reactivation and subsequent alanine transaminase (ALT) elevation of more than threefold the upper limit of normal^20^.

#### Cohort 2

We recruited non-HCV-infected patients with resolved HBV infection who ((1) either did or did not have HBV reactivation during immunosuppressive or ani-cancer therapy, (2) had preserved serum prior to treatment, and (3) were from Hokkaido University Hospital or Teine Keijinkai Hospital between December 2008 and June 2020. Patients without HBV reactivation were followed up for at least one year and monitored for HBV-DNA every 3 months. Additionally, we recruited non-HCV-infected patients with resolved HBV infection who (1) either did or did not have HBV reactivation after bone marrow transplantation (BMT), (2) had preserved serum prior to transplantation, and (3) were from Hokkaido University Hospital. We compared the cytokine levels and clinical factors between patients with or without HBV reactivation and analyzed the similarities and differences among patients receiving DAAs, anti-cancer/immunosuppressive therapy, or BMT. This study conformed to the Declaration of Helsinki. Hokkaido University Hospital and each ethical committee of the participating institutes approved the study protocol (Approval numbers 016-0059 and 020-0015). Enrolled patients provided written informed consent to participate in this study or did not decline to participate in this study. The ethics committee specifically approved the non-decline option being included in the study in lieu of written informed consent for some patients.

### Analysis of serum cytokines

Serum cytokines, including IL-1α, IL-1β, IL-4, IL-6, IL-8, IL-10, IL-13, monocyte chemotactic protein (MCP)-1, IFN-γ, and tumor necrosis factor α (TNFα), were analyzed using Human Inflammation Antibody Array 1 (RayBiotech Life, Inc., GA, USA) according to the manufacturer’s protocol. We selected this cytokine array based on previous reports, indicating cytokines that could affect HBV replication and lifecycle and contribute to HBV clearance^15,17,18^.

### Examination of HBV markers

Serum titers of HBsAg and anti-HBs were measured by chemiluminescence immunoassays using the Architect HBsAg-QT assay and Architect anti-HBs assay (Abbott, Tokyo, Japan), and the lower-limits of detection were 0.05 IU mL^−1^ and 10 mIU mL^−1^, respectively. Anti-HBc was measured by chemiluminescence immunoassays using the Architect Anti-HBc assay (Abbott, Japan), with a cutoff value of 1^21,22^. Serum HBV-DNA level was analyzed by TaqMan polymerase chain reaction (PCR) assay (Cobas AmpliPrep/Cobas TaqMan HBV test, v2.0), and the quantitative range was 1.3–8.3 log_10_ IU mL^−1^.

### Association of IL-1β single nucleotide polymorphism genotyping.

In this study, obtained genomic DNA from 50 patients. To analyze IL-1β rs16944 (A/G), we utilized a TaqMan single nucleotide polymorphism (SNP) genotyping kit (Applied Biosystems, Foster City, CA, USA). We conducted IL-1β genotyping according to the manufacturer's protocol, as described previously^23^.

### Statistical analyses

Continuous variables were analyzed using the Mann–Whitney U test, paired t-test, or analysis of variance. Categorical data were analyzed using Fisher’s exact test. Multivariate logistic regression analysis with stepwise forward selection was performed for the cytokines identified as significant factors (*P* ≤ 0.05) in the univariate analyses of factors associated with HBV reactivation during DAA treatment. *P*-values were two-tailed and were set statistically significant at *P* < 0.05. All statistical analyses were conducted using SPSS (version 24.0; IBM Japan, Tokyo, Japan).

## Results

### Patient characteristics

We included 11 patients who experienced HBV reactivation or reappearance during and after DAA and had proper preserved serum^5,24^. Between April 2014 and May 2020, a total of 411 HCV-infected patients with resolved HBV infection were analyzed; from these, 11 (2.7%) experienced HBV reactivation or reappearance during DAA treatment. The characteristics of these 11 patients are shown in Table 1 and Supplementary Table S1.

**Table 1:** Baseline characteristics of HCV-infected patients with or without resolved HBV infection.

	Reactivation (R)	Non-reactivation (N-R)	HCV control	Univariate analysis	Analysis of variance
Number	11	35	19	P value (R versus N-R)	P value (ALL)
Age (years)^a^	69 (63–75)	69 (44–87)	63 (33–83)	0.939	0.053
Sex (male/female)	4/7	16/19	5/14	0.425	0.371
HCV genotype (1/2)	8/3	25/10	13/6	0.628	0.962
HCV-RNA (log IU/mL)^a^	6.2 (4.3–7.1)	6.3 (4.7–6.9)	6.4 (4.2–7.2)	0.761	0.535
DCV/ASV, SOF/LDV, SOF/RBV	3/5/3	7/18/10	4/9/6	0.874	0.987
Platelet count (× 10^4^)^a^	16.4 (5.9–21.1)	15.8 (5.4–27.7)	15.7 (5.8–24.3)	0.594	0.683
Albumin (g/dL)^a^	3.98 (3.3–4.7)	4.1 (2.8–5.0)	4.2 (3.3–4.8)	0.461	0.602
AST (IU/L)^a^	48 (33–205)	39 (22–106)	31 (16–143)	0.183	0.299
ALT (IU/L)^a^	46 (22–173)	41 (13–134)	31 (6–151)	0.576	0.249
γGTP (IU/L)^a^	31 (18–104)	31 (12–276)	29 (11–99)	0.722	0.709
FIB-4 index^a^	2.61 (1.71–16.53)	1.62 (0.28–15.98)	2.9 (0.59–6.27)	0.117	0.28
**HBV status**					
Anti-HBs±	3/8	19/16		0.111	–
Anti-HBs negative or < 30 mIU/mL	0/11	12/23		*0.021	

*HCV* hepatitis C virus; *HBV* hepatitis B virus, *HCC* hepatocellular carcinoma, *AST* aspartate aminotransferase, *ALT* alanine aminotransferase, *γGTP* γ-glutamyl transpeptidase, *AFP* a-fetoprotein, *anti-HBs* antibody to hepatitis B surface antigen.

*Statistically significant difference, P < 0.05.

^a^Data are shown as median (range) values.

In addition, a total of 79 HCV infected patients with resolved HBV infection received DAAs between October 2014 and May 2016 at Hokkaido University Hospital. Of those, we recruited 35 consecutive patients who had proper clinical information, preserved serum at baseline, and EOT as controls. Additionally, we collected information regarding preserved serum at baseline and at EOT from 19 consecutive HCV-infected patients without resolved or present HBV infection who received IFN-free DAAs between 2014 and 2016 at Hokkaido University Hospital.

The baseline patient characteristics are shown in Table 1. The serum cytokine levels of 65 HCV-infected patients who were treated with IFN-free DAAs were analyzed. Comparison among the study groups (HCV-infected patients with resolved HBV infection and reactivation/reappearance, HCV-infected patients with resolved HBV infection and no reactivation/reappearance, HCV-infected patients without resolved HBV infection) showed that baseline patient characteristics were similar among the groups.

A comparison of anti-HBs titers between patients with resolved HBV infection with HBV reactivation/reappearance during DAA therapy and those without HBV reactivation/reappearance during DAA therapy revealed that the rate of a negative anti-HBs titer or an anti-HBs titer of < 30 mIU/mL was significantly higher in the former than in the latter.

### Comparison of baseline cytokine levels among HCV-infected patients with resolved HBV infection and reactivation/reappearance or non-reactivation/reappearance during DAA therapy

**Figure 1:**
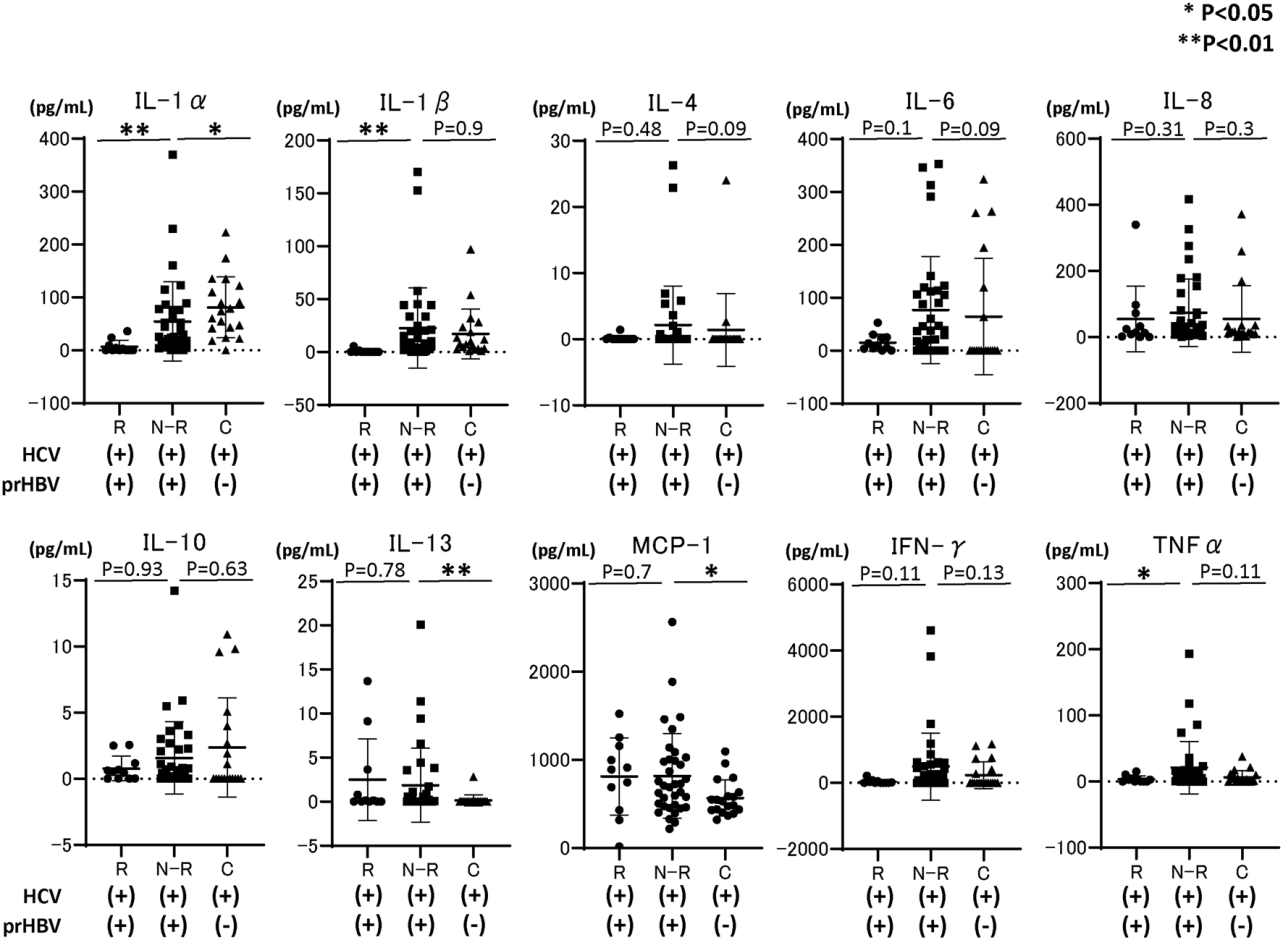
Comparison of baseline cytokine levels among HCV-infected patients with previous HBV infection, those with or without HBV reactivation during DAA therapy, and HCV-infected patients without previous HBV infection. Each data are shown as median ± SD. *P < 0.05, ** P < 0.01. *HCV* hepatitis C virus, *HBV* hepatitis B virus, *DAA* direct-acting antiviral, *IL* interleukin, *MCP* monocyte chemotactic protein, *IFN* interferon, *TNF* tumor necrosis factor, *SD* standard deviation, *R* HBV reactivation, *N-R* non-HBV reactivation, *C* HCV control without previous HBV infection, *prHBV* previously resolved HBV infection.

**Figure 2:**
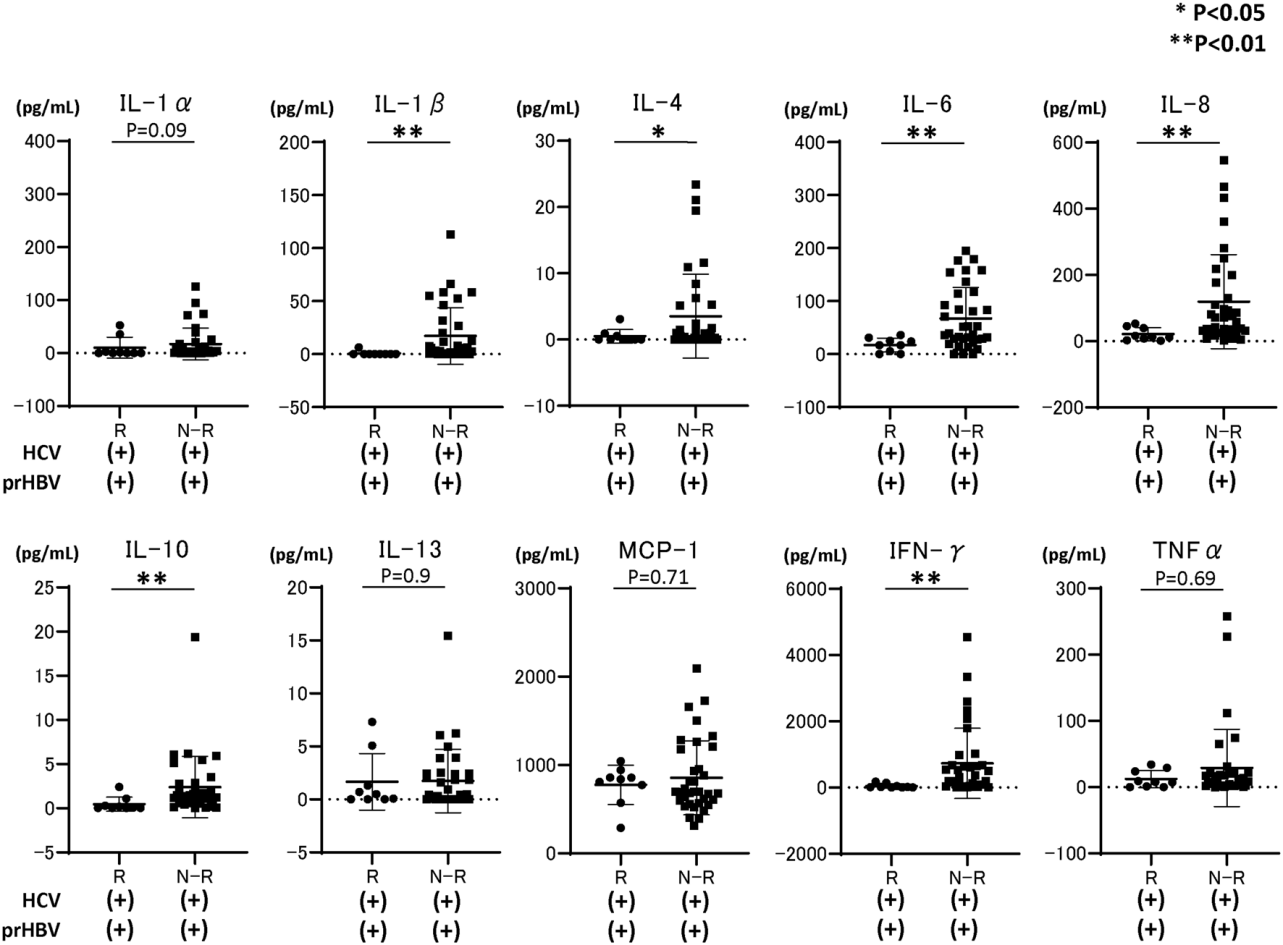
Comparison of cytokine levels at the end of DAA treatment among HCV-infected patients without previous HBV infection, those with or without HBV reactivation during DAA therapy, and HCV-infected patients without previous HBV infection. Each data are shown as median ± SD. *P < 0.05, **P < 0.01. *HCV* hepatitis C virus, *HBV* hepatitis B virus, *DAA* direct-acting antiviral, *IL* interleukin, *MCP* monocyte chemotactic protein, *IFN* interferon, *TNF* tumor necrosis factor, *SD* standard deviation, *R* HBV reactivation, *N-R* non-HBV reactivation, *prHBV* previously resolved HBV infection.

We subsequently compared the baseline cytokine levels, including IL-1α, IL-1β, IL-4, IL-6, IL-8, IL-10, IL-13, MCP-1, IFN-γ, and TNFα, among the study groups. As shown in Fig. 1, baseline IL-1α, IL-1β, and TNFα levels in patients with HBV reactivation or reappearance were significantly lower than those in patients without HBV reactivation or reappearance during DAA therapy. Subsequently, we compared the cytokine levels at EOT between patients with (n = 9) and without (n = 35) HBV reactivation or reappearance during DAA therapy. As shown in Fig. 2, several cytokines, including IL-1β and IFN-γ, were significantly lower in patients with HBV reactivation or reappearance than in patients without HBV reactivation or reappearance during DAA therapy.

### Multivariate analysis of the association between baseline cytokine levels and HBV reactivation during DAA therapy

We conducted multivariate logistic regression analysis of cytokines significantly associated with HBV reactivation (P ≤ 0.05) at baseline in the univariate analysis (i.e., IL-1α, IL-1β, and TNFα). The best cutoff values for IL-1α, IL-1β, and TNFα were determined by receiver operating characteristics (ROC) analysis. As shown in Table 2, the IL-1β level (odds ratio 47.6, 95% confidence interval [CI], 6.94–333.3; P = 0.001) was significantly associated with HBV reactivation or reappearance during the DAA therapy. In addition, we performed multivariate logistic regression analysis of cytokines and clinical factors that showed a significant association with HBV reactivation (P ≤ 0.05) at baseline in the univariate analysis (i.e., IL-1α and IL-1β, TNFα, and anti-HBs titer). The results revealed that IL-1β (odds ratio 47.6, 95% CI 6.94–333.3; P < 0.001) was significantly associated with HBV reactivation or reappearance during DAA therapy (Supplementary Table S2).

**Table 2:** Univariate and multivariate logistic regression analyses of cytokines significantly associated with HBV reactivation.

	Reactivation	Non-reactivation	Univariate analysis	Multivariate analysis	Odds ratio
Number	11	35			
**IL-1α (pg/mL)**					
< 10.22, 10.22 ≤	9/2	9/26	*0.001	0.202	
**IL-1β (pg/mL)**					
< 0.18, 0.18 ≤	9/2	3/32	* < 0.001	*0.001	47.6 (6.94–333.3)
**TNFα (pg/mL)**					
< 4.813, 4.813 ≤	9/2	13/22	*0.01	0.111	

*HBV* hepatitis B virus, *IL* interleukin, *TNF* tumor necrosis factor.

*Statistically significant difference, P < 0.05.

**Figure 3:**
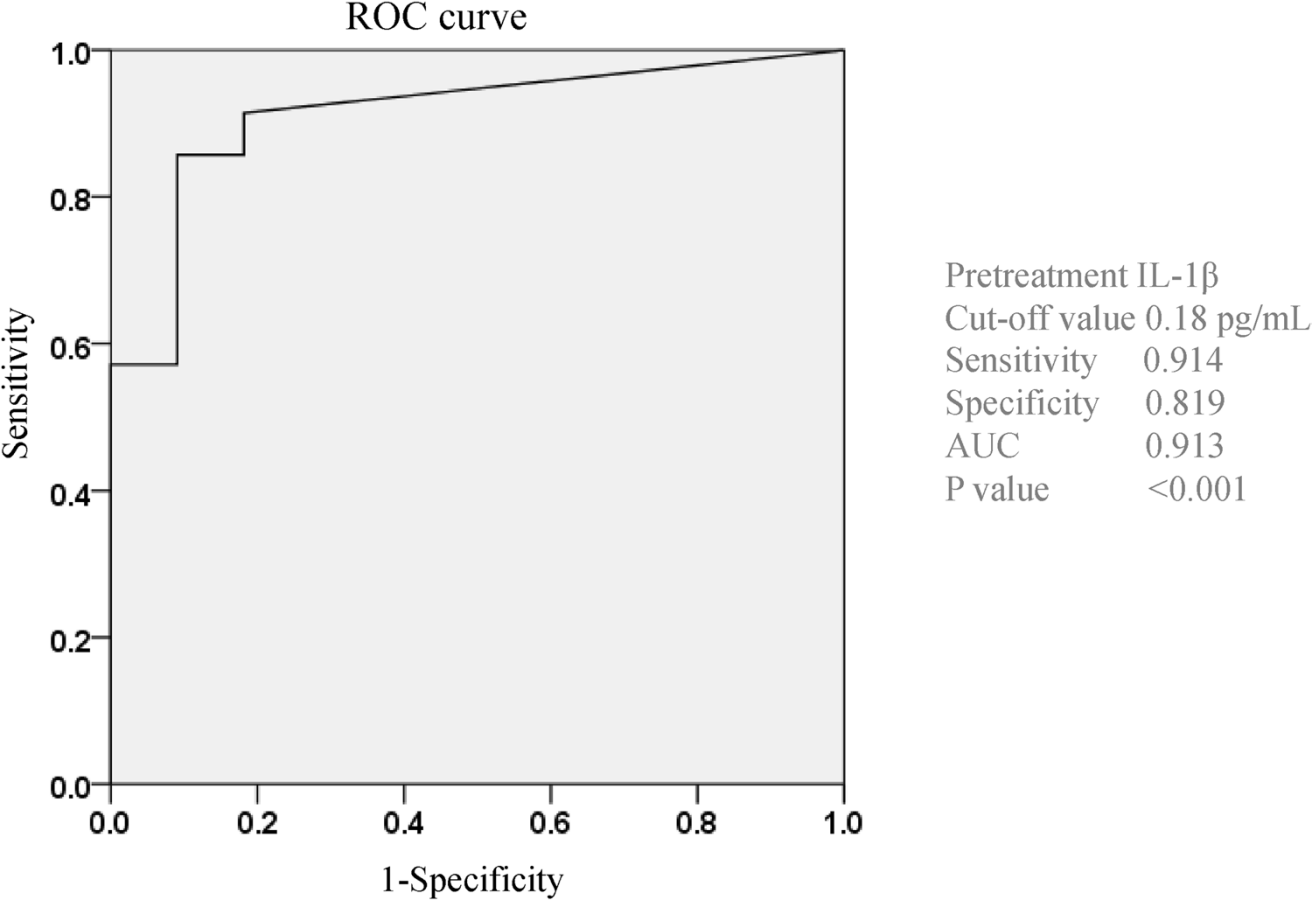
Cutoff value of baseline IL-1β level for predicting HBV reactivation in HCV-infected patients with previous HBV infection during DAA therapy. Receiver operating characteristic (ROC) curve analysis for baseline IL-β levels in HCV-infected patients with previous HBV infection. The cutoff baseline IL-1β level for predicting HBV reactivation during DAA therapy was set at 0.18 pg/mL (area under the ROC curve, 0.913; sensitivity, 0.914; specificity, 0.819). *HCV* hepatitis C virus, *HBV* hepatitis B virus, *DAA* direct-acting antiviral, *IL* interleukin.

As shown in Fig. 3, the cutoff value of IL-1β to predict HBV reactivation was set at 0.18 pg/mL, and the sensitivity, specificity, and area under the ROC curve for predicting HBV reactivation were 0.914, 0.819, and 0.913, respectively.

### Association between the baseline serum IL-1β level and the IL-1β rs16944 SNP among patients with previous HBV infection, those with or without HBV reactivation or reappearance during DAA therapy, and HCV-infected patients without resolved HBV infection

**Figure 4:**
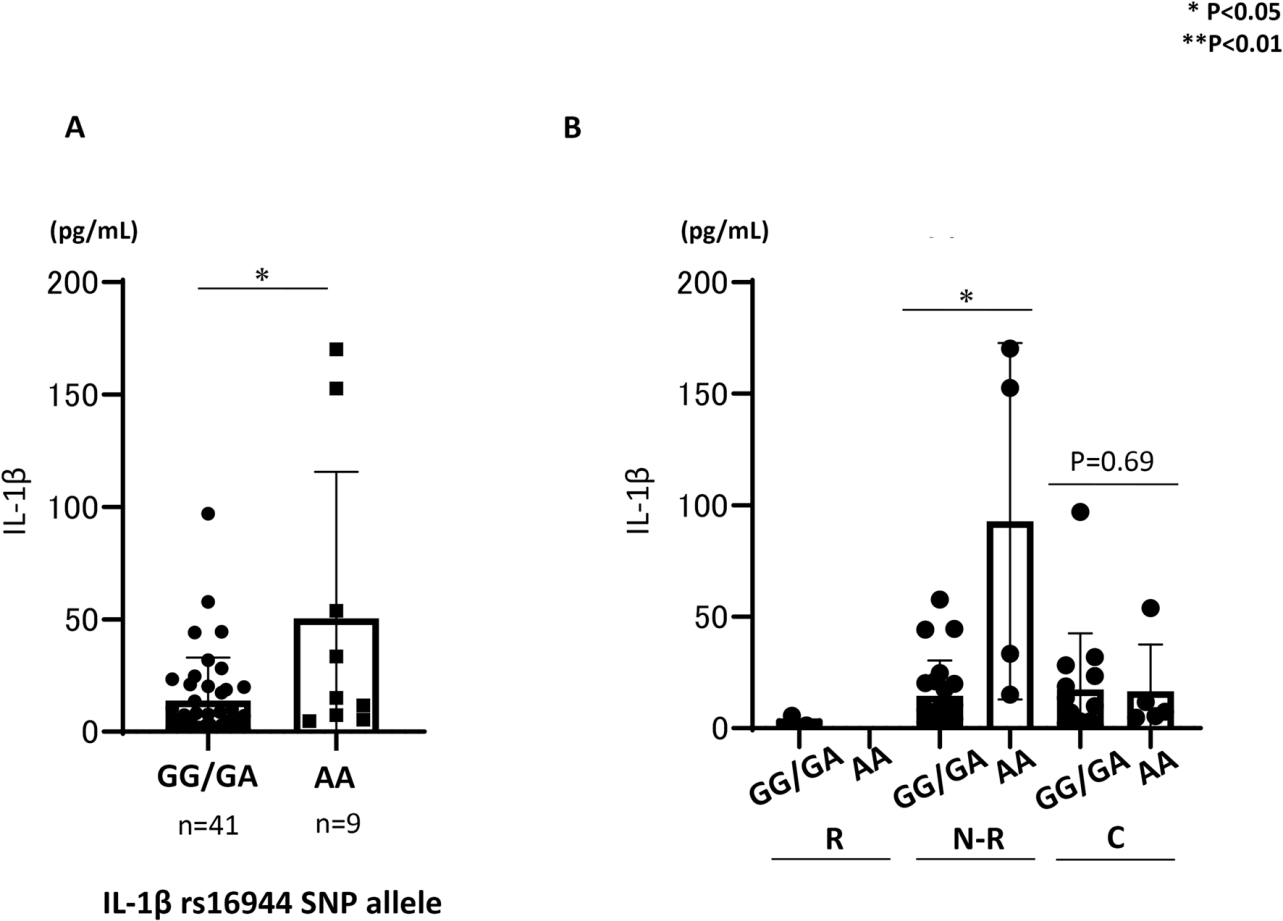
Comparison of IL-1β levels in HCV-infected patients treated with DAAs stratified according to the presence of the IL-1β rs16944 single nucleotide polymorphism (SNP). (**A**) Comparison of baseline IL-1β levels between HCV-infected patients with and without the IL-1β rs16944 SNP AA allele. (**B**) Comparison of baseline IL-1β levels among HCV-infected patients treated with DAAs stratified according to the presence of the IL-1β rs16944 SNP, existence of previous HBV infection, and presence of HBV reactivation during DAA therapy. *HCV* hepatitis C virus, *HBV* hepatitis B virus, *DAA* direct-acting antiviral, *IL* interleukin, *R* HBV reactivation, *N-R* non-HBV reactivation, *C* HCV control without previous HBV infection.

To investigate the factors associated with baseline serum IL-1β levels that are possibly involved in HBV reactivation or reappearance during DAA treatment, we analyzed the association between the baseline serum IL-1β level and the IL-1β rs16944 SNP in patients with previous HBV infection, those with or without HBV reactivation or reappearance during DAA therapy, and HCV-infected patients without resolved HBV infection. The IL-1β rs16944 SNP is reported to be associated with serum IL-1β levels^25^. Figure 4A shows the association between baseline serum IL-1β levels and the IL-1β rs16944 SNP in HCV-infected patients with or without resolved HBV infection (n = 50). Baseline IL-1β levels were significantly higher in patients with the IL-1β rs16944 SNP AA allele than in those with IL-1β non-AA allele. Figure 4B shows the serum IL-1β levels according to the IL-1β rs16944 SNP, existence of resolved HBV infection, and HBV reactivation or reappearance during DAA treatment. As shown in Fig. 4B, in patients with HBV reactivation, no patient had the IL-1β rs16944 SNP AA allele. In patients with resolved HBV infection and those without HBV reactivation during DAA treatment, patients with the IL-1β AA allele had significantly higher IL-1β levels than those without the non-AA allele. In patients without resolved HBV infection, baseline IL-1β levels were similar between patients with the IL-1β rs16944 SNP AA allele and non-AA allele.

### Association between baseline cytokine levels and de novo HBV reactivation during immunosuppressive therapy/chemotherapy or BMT in non-HCV-infected patients with previous HBV infection

Finally, we compared the cytokine levels among non-HCV-infected patients with or without HBV reactivation in patients receiving anti-cancer/immunosuppressive therapy or BMT.

**Figure 5:**
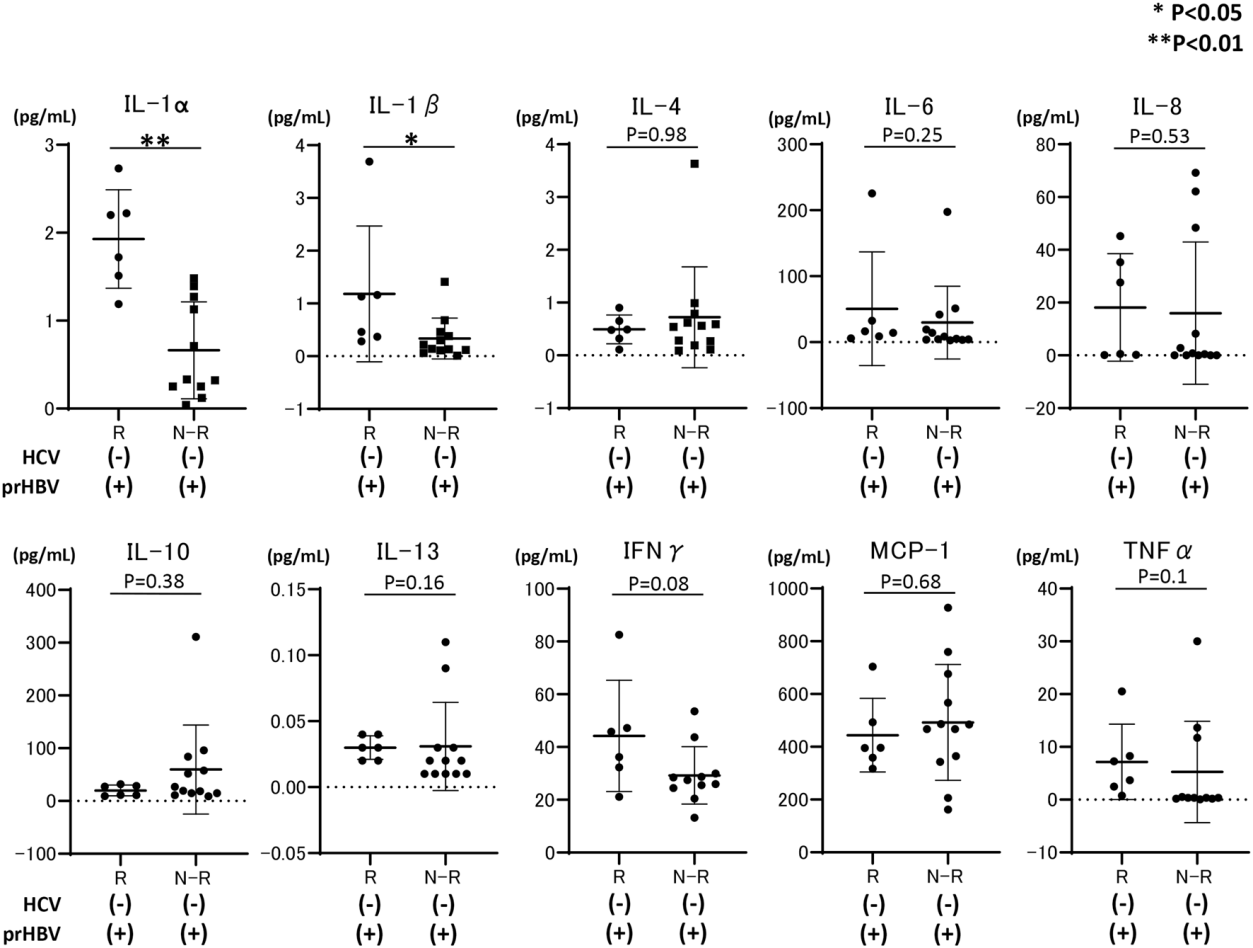
Comparison of baseline cytokine levels among HCV-non-infected patients with previous HBV infection with or without HBV reactivation during anti-cancer/immunosuppressive therapy or bone marrow transplantation. Each data are shown as median ± SD. *P < 0.05, **P < 0.01. *HCV* hepatitis C virus, *HBV* hepatitis B virus, *IL* interleukin, *MCP* monocyte chemotactic protein, *IFN* interferon, *TNF* tumor necrosis factor, *SD* standard deviation, *R* HBV reactivation, *N-R* non-HBV reactivation, *prHBV* previously resolved HBV infection.

**Figure 6:**
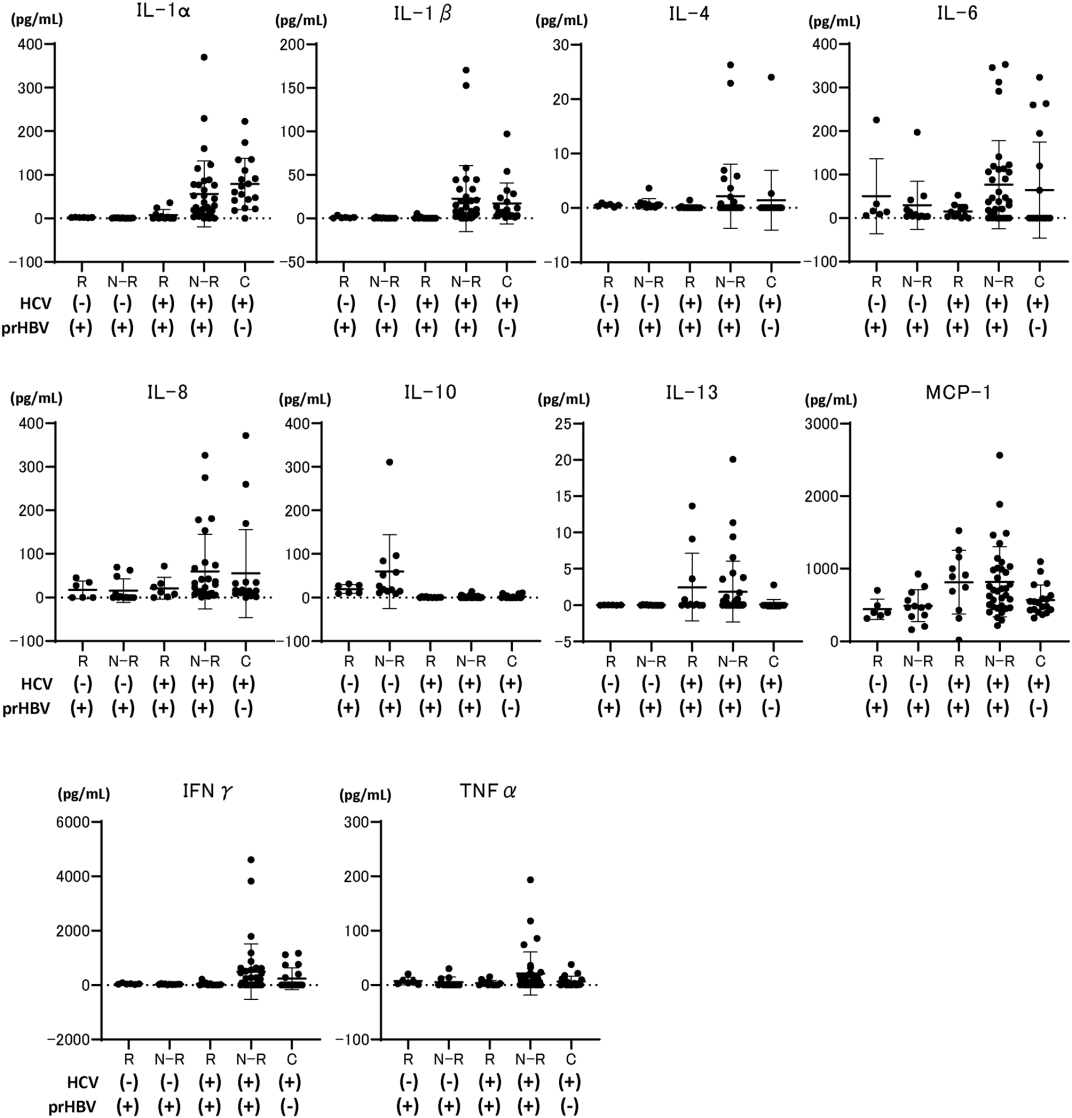
Comparison of baseline cytokine levels among patients classified according to the presence of HCV infection, existence of previous HBV infection, and presence of HBV reactivation during DAA, anti-cancer/immunosuppressive therapy, or bone marrow transplantation. Each data are shown as median ± SD. *P < 0.05, **P < 0.01. *HCV* hepatitis C virus, *HBV* hepatitis B virus, *DAA* direct-acting antiviral, *IL* interleukin, *MCP* monocyte chemotactic protein, *IFN* interferon, *TNF* tumor necrosis factor, *SD* standard deviation, *R* HBV reactivation, *N-R* non-HBV reactivation, *C* HCV control without previous HBV infection, *prHBV* previously resolved HBV infection.

Conversely to HCV-infected patients with resolved HBV infection, non-HCV-infected patients with HBV reactivation during anti-cancer/immunosuppressive therapy or BMT had significantly higher IL-1α and -1β levels than did those without HBV reactivation (Fig. 5 and Supplementary Table S3). A subgroup analysis stratified by receiving or not receiving BMT is shown in Supplementary Fig. S1. However, as shown in the landscape of cytokine levels in all groups (including HCV-infected or non-HCV-infected patients) (Fig. 6), HCV-infected patients had remarkably higher IL-1β levels than did others. HCV-infected patients with HBV reactivation during DAA therapy had remarkably lower IL-1β levels than did other HCV-infected patients.

## Discussion

In this study, by comparing cytokine levels among HCV-infected patients with resolved HBV infection and HBV reactivation or reappearance during DAA therapy, HCV-infected patients with resolved HBV infection and no HBV reactivation or reappearance during DAA therapy, and HCV-infected patients without resolved HBV infection, we identified that low baseline serum IL-1β level is predictive of de novo HBV reactivation during DAA therapy (odds ratio 47.6, 95% CI 6.94–333.3). HCV-infected patients with the IL-1β rs16944 SNP AA allele had significantly higher IL-1β levels, and no HCV-infected patient with the IL-1β rs16944 SNP AA allele experienced HBV reactivation during DAA therapy. Conversely in HCV-infected patients with resolved HBV infection, non-HCV infected patients with HBV reactivation during anti-cancer/immunosuppressive therapy or BMT had significantly higher IL-1β levels than did those without HBV reactivation. However, the comparison of cytokine levels in all groups, including patients with or without HCV infection, revealed that HCV-infected patients had remarkably higher IL-1β levels than did others. HCV-infected patients with HBV reactivation during DAA therapy had remarkably lower IL-1β levels than did other HCV-infected patients, demonstrating the mechanical insight of de novo HBV reactivation during DAA therapy and different mechanisms of de novo HBV reactivation between anti-cancer therapy/immunosuppressive therapy and DAAs. In addition, evaluating baseline IL-1β prior to DAA treatment for HCV-infected patients with resolved HBV infection is a potential method for predicting de novo HBV reactivation during DAA treatment. The mechanical insight of de novo HBV reactivation during DAA therapy for HCV-infected patients with resolved HBV infection has not been fully elucidated. A possible hypothesis is that any suppressive effect of HBV replication is lost by DAA therapy, resulting in HBV reactivation. Previous reports have shown that in patients with HBV/HCV co-infection, HCV is usually thought to suppress HBV replication, resulting in HCV becoming the dominant virus in these patients^26,27^. Thus, we hypothesized that some factors induced by HCV infection, such as IFN, might be high in patients with HBV reactivation, and HCV eradication by DAAs might lead to a decrease in factors that could suppress HBV replication. However, as shown in Fig. 1, cytokine arrays demonstrated that in HCV-infected patients with HBV reactivation or reappearance during DAA therapy, the IL-1α, IL1-β, and TNF-α levels were significantly and remarkably lower than those in HCV-infected patients without HBV reactivation. Multivariate analysis revealed that baseline serum IL-1β was significantly associated with HBV reactivation or reappearance during DAA therapy. Watashi et al. reported that, among cytokines and chemokines, IL-1β and TNFα strongly suppressed hepatocyte susceptibility to HBV infection through activation-induced cytidine deaminase upregulation^28^. Thus, at baseline, HCV-infected patients with HBV reactivation during DAA therapy can easily experience HBV reactivation because the strong suppressive cytokine level of IL-1β was quite low. Additionally, in vitro and in vivo analyses have revealed that HCV protein could suppress HBV replication^29,30^. Therefore, DAA therapy clearing the intracellular HCV protein might trigger HBV reactivation during DAA therapy in HCV-infected patients with low baseline IL-1β levels. Notably, analysis at EOT (Fig. 2) revealed that the IL-1β level was still significantly higher in patients without HBV reactivation than in those with HBV reactivation during DAA therapy; thus, patients without HBV reactivation might have a cytokine environment that suppresses HBV replication. The reason why some HCV-infected patients with resolved HBV infection had lower baseline IL-1β levels has not been clarified well. Thus far, it has been reported that several IL-1β SNPs might affect HBV infection^31,32^. Among these, the IL-1β rs16944 AA SNP is reported to be directly associated with high serum IL-1α levels in patients with HBV infection^25^. Therefore, in this study, we focused on the IL-1β rs16944 SNP, which is reported to be associated with serum IL-1β levels^25^. As shown in Fig. 4A, in HCV-infected patients with IL-1β rs16944, those with the SNP AA allele had significantly higher serum IL-1β levels. Among patients classified according to the presence of resolved HBV infection, HBV reactivation during DAA therapy, and IL-1β allele (1) in HCV-infected patients with HBV reactivation during DAA treatment, no patient had IL-1β rs16944 AA allele, and (2) in HCV-infected patients without HBV reactivation during DAA therapy, IL-1β levels were significantly higher in patients with the IL-1β rs16944 SNP AA allele than in those with the non-AA allele. However, these findings were not observed in HCV-infected patients without resolved HBV infection. Thus, in patients with the IL-1β rs16944 SNP AA allele, the risk of HBV reactivation during DAA treatment might be lower. However, it is unclear why the serum IL-1β level was elevated only in HCV-infected patients with resolved HBV infection and the IL-1β rs16944 SNP AA allele, but not in HCV-infected patients without resolved HBV infection and the IL-1β rs16944 SNP AA allele. One explanation is the small number of included patients, thus further analysis is required.

In this study, we also analyzed the relationship between HBV reactivation and baseline cytokine levels in non-HCV-infected patients with resolved HBV infection who were treated with immunosuppressive agents/anti-cancer drugs or underwent BMT. As shown in Fig. 5, compared to HCV-infected patients with HBV reactivation, non-HCV infected patients with HBV reactivation who were treated with immunosuppressive agents/anti-cancer drugs or underwent BMT had significantly higher IL-1β levels than did those without HBV reactivation. Thus, the involvement of cytokines in HBV reactivation during DAA treatment, immunosuppressive and anti-cancer therapy, or BMT might be different.

This study has some limitations. First was the retrospective study design, which resulted in some missing information. Second was the relatively small number of patients. In particular, the number of patients with HBV reactivation who underwent analysis of the IL-1b SNP was limited. This should be considered when interpreting the results. Third, the follow-up period was limited. Therefore, further analysis is required.

In conclusion, low baseline serum IL-1 β levels predict de novo HBV reactivation during DAA therapy, but not during anti-cancer/immunosuppressive therapy, for HCV-infected patients with resolved HBV infection.

## Supplementary Information

Supplementary Information 1.

Supplementary Information 2.

Supplementary Information 3.

## Data Availability

All relevant data are within the manuscript and its supplementary material.

## References

[1] Suda G, Sakamoto N (2021). Recent advances in the treatment of hepatitis C virus infection for special populations and remaining problems. J. Gastroenterol. Hepatol..

[2] Suda G, Ogawa K, Morikawa K, Sakamoto N (2018). Treatment of hepatitis C in special populations. J. Gastroenterol..

[3] Suda G (2018). Daclatasvir and asunaprevir in hemodialysis patients with hepatitis C virus infection: A nationwide retrospective study in Japan. J. Gastroenterol..

[4] Curry MP (2015). Sofosbuvir and Velpatasvir for HCV in patients with decompensated cirrhosis. N. Engl. J. Med..

[5] Kawagishi N (2017). Comparing the risk of hepatitis B virus reactivation between direct-acting antiviral therapies and interferon-based therapies for hepatitis C. J. Viral Hepat..

[6] Collins JM (2015). Hepatitis B virus reactivation during successful treatment of hepatitis C virus with sofosbuvir and simeprevir. Clin. Infect. Dis..

[7] Kawagishi N (2017). Hepatitis B virus reactivation during hepatitis C direct-acting antiviral therapy in patients with previous HBV infection. J. Hepatol..

[8] Takayama H, Sato T, Ikeda F, Fujiki S (2016). Reactivation of hepatitis B virus during interferon-free therapy with daclatasvir and asunaprevir in patient with hepatitis B virus/hepatitis C virus co-infection. Hepatol. Res..

[9] Di Bisceglie AM (2015). Recent US Food and Drug Administration warnings on hepatitis B reactivation with immune-suppressing and anticancer drugs: Just the tip of the iceberg?. Hepatology.

[10] Umemura T, Tanaka E, Kiyosawa K, Kumada H, Japan de novo Hepatitis, B. R. G (2008). Mortality secondary to fulminant hepatic failure in patients with prior resolution of hepatitis B virus infection in Japan. Clin. Infect. Dis..

[11] Hui CK (2006). Kinetics and risk of de novo hepatitis B infection in HBsAg-negative patients undergoing cytotoxic chemotherapy. Gastroenterology.

[12] Suda T (2017). Reactivation of hepatitis B virus from an isolated anti-HBc positive patient after eradication of hepatitis C virus with direct-acting antiviral agents. J. Hepatol..

[13] Hayashi K (2016). A case of acute hepatitis B in a chronic hepatitis C patient after daclatasvir and asunaprevir combination therapy: Hepatitis B virus reactivation or acute self-limited hepatitis?. Clin. J. Gastroenterol..

[14] De Monte A (2016). Direct-acting antiviral treatment in adults infected with hepatitis C virus: Reactivation of hepatitis B virus coinfection as a further challenge. J. Clin. Virol..

[15] Xia Y, Protzer U (2017). Control of hepatitis B virus by cytokines. Viruses.

[16] Isorce N (2016). Antiviral activity of various interferons and pro-inflammatory cytokines in non-transformed cultured hepatocytes infected with hepatitis B virus. Antiviral Res..

[17] Hong MH (2012). Transforming growth factor-beta1 suppresses hepatitis B virus replication by the reduction of hepatocyte nuclear factor-4alpha expression. PLoS ONE.

[18] Hosel M (2009). Not interferon, but interleukin-6 controls early gene expression in hepatitis B virus infection. Hepatology.

[19] Mochida S (2016). Nationwide prospective and retrospective surveys for hepatitis B virus reactivation during immunosuppressive therapies. J. Gastroenterol..

[20] Hsu C (2014). Chemotherapy-induced hepatitis B reactivation in lymphoma patients with resolved HBV infection: A prospective study. Hepatology.

[21] Suzuki K (2021). Tenofovir-disoproxil-fumarate modulates lipid metabolism via hepatic CD36/PPAR-alpha activation in hepatitis B virus infection. J. Gastroenterol..

[22] Suzuki K (2019). Entecavir treatment of hepatitis B virus-infected patients with severe renal impairment and those on hemodialysis. Hepatol. Res..

[23] Kawagishi N (2018). Liver steatosis and dyslipidemia after HCV eradication by direct acting antiviral agents are synergistic risks of atherosclerosis. PLoS ONE.

[24] Doi A (2017). Frequency of, and factors associated with, hepatitis B virus reactivation in hepatitis C patients treated with all-oral direct-acting antivirals: Analysis of a Japanese prospective cohort. Hepatol. Res..

[25] Wu JF (2018). Clinical predictors of liver fibrosis in patients with chronic hepatitis B virus infection from children to adults. J. Infect. Dis..

[26] Raimondo G, Cacciamo G, Saitta C (2005). Hepatitis B virus and hepatitis C virus co-infection: Additive players in chronic liver disease?. Ann. Hepatol..

[27] Chu CM, Yeh CT, Liaw YF (1998). Low-level viremia and intracellular expression of hepatitis B surface antigen (HBsAg) in HBsAg carriers with concurrent hepatitis C virus infection. J. Clin. Microbiol..

[28] Watashi K (2013). Interleukin-1 and tumor necrosis factor-alpha trigger restriction of hepatitis B virus infection via a cytidine deaminase activation-induced cytidine deaminase (AID). J. Biol. Chem..

[29] Shih CM, Lo SJ, Miyamura T, Chen SY, Lee YH (1993). Suppression of hepatitis B virus expression and replication by hepatitis C virus core protein in HuH-7 cells. J. Virol..

[30] Zhu W (2012). Inhibition of the HCV core protein on the immune response to HBV surface antigen and on HBV gene expression and replication in vivo. PLoS ONE.

[31] Tuncbilek S (2014). Relationship between cytokine gene polymorphisms and chronic hepatitis B virus infection. World J. Gastroenterol..

[32] Hirankarn N, Kimkong I, Kummee P, Tangkijvanich P, Poovorawan Y (2006). Interleukin-1beta gene polymorphism associated with hepatocellular carcinoma in hepatitis B virus infection. World J. Gastroenterol..

