# Supplementary figures and images for: Serum IL-1β predicts de novo hepatitis B virus reactivation during direct-acting antiviral therapy for hepatitis C, not during anti-cancer/immunosuppressive therapy
Source: Sci Rep. 2022 Oct 7;12:16800. doi: 10.1038/s41598-022-21315-z (PMC9546937; doi:10.1038/s41598-022-21315-z)

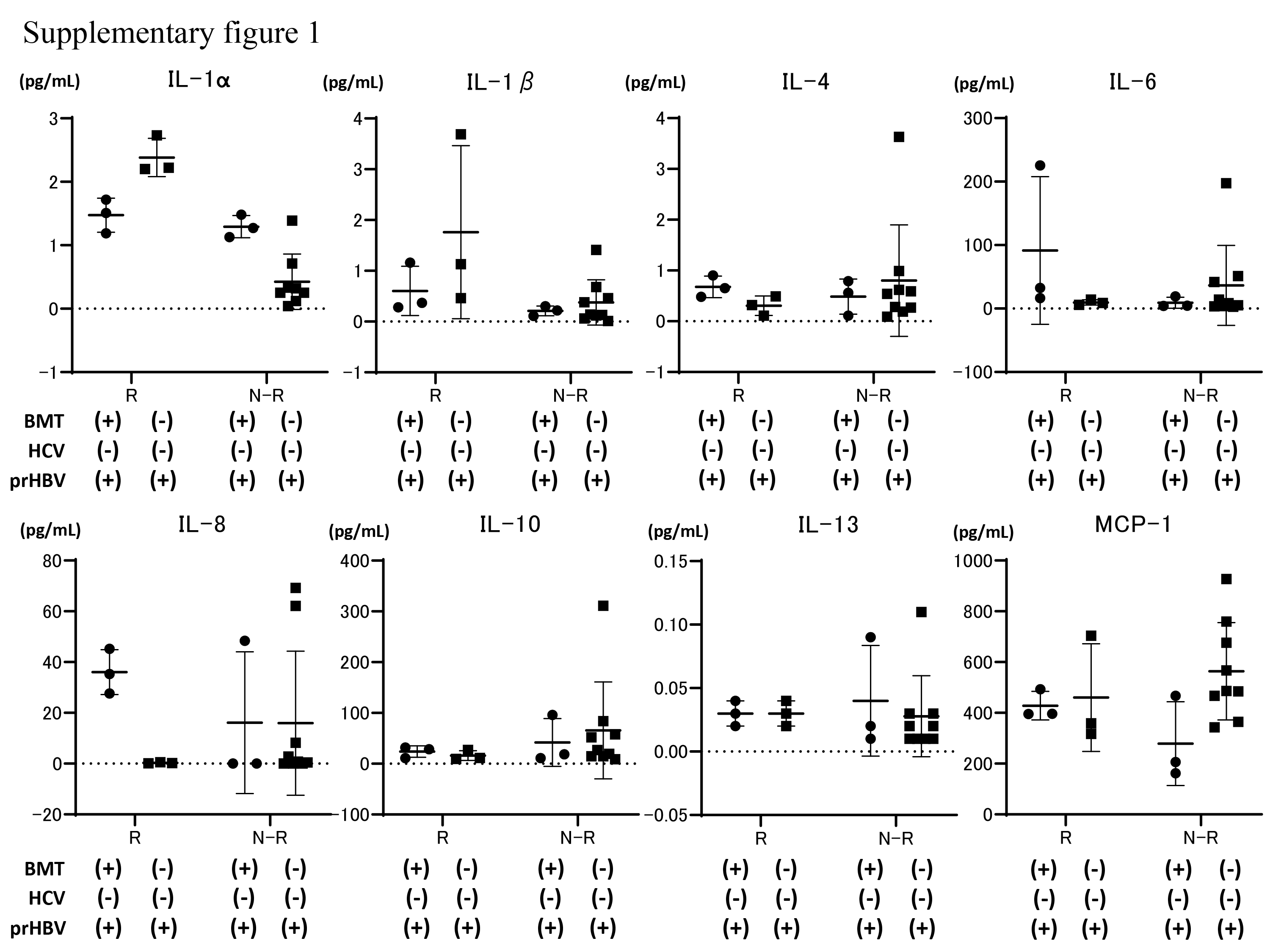

Supplement: Supplementary file 1 — Supplementary Information 1. [file 41598_2022_21315_MOESM1_ESM.tif]

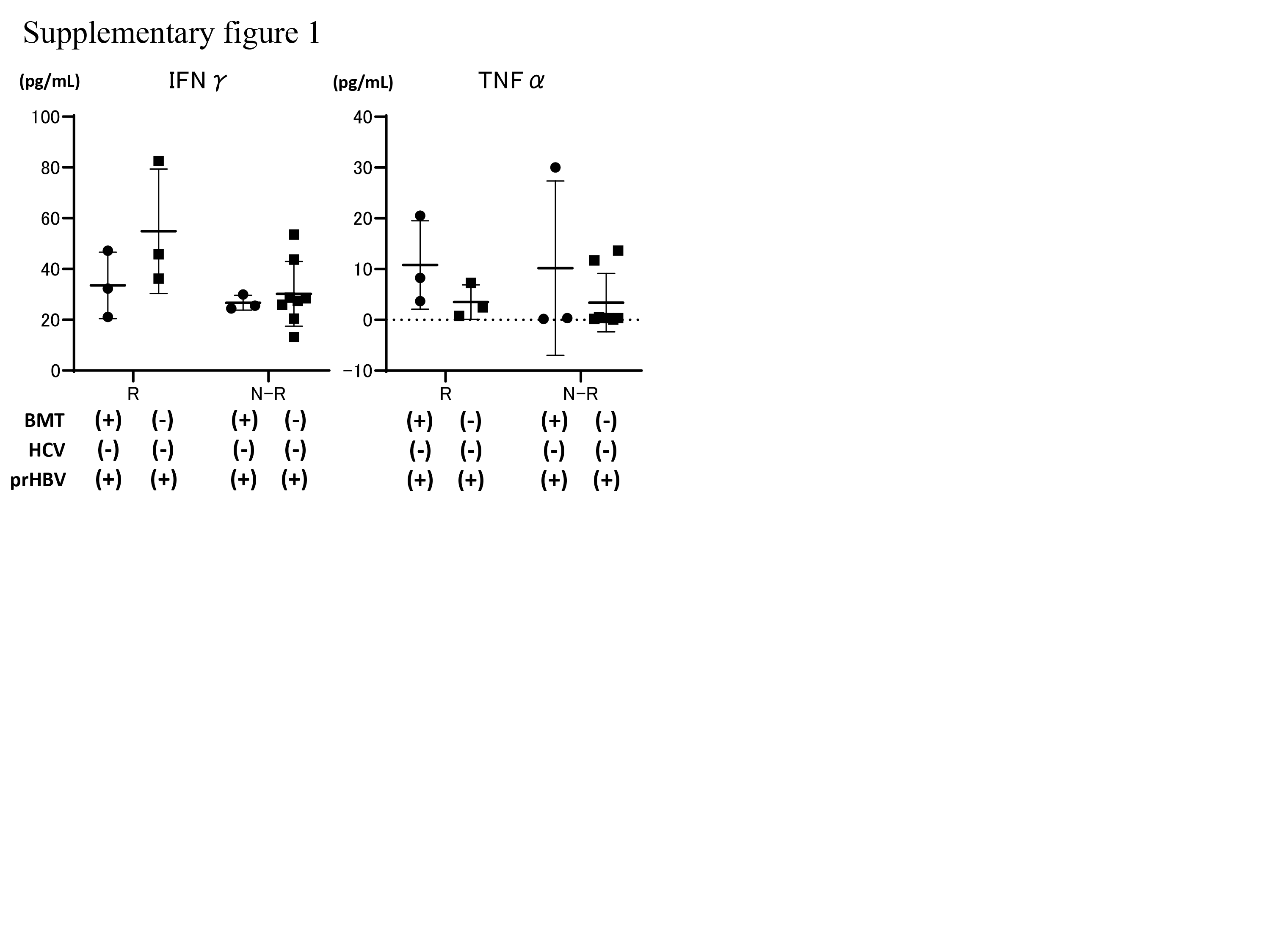

Supplement: Supplementary file 2 — Supplementary Information 2. [file 41598_2022_21315_MOESM2_ESM.tif]
