# Supplementary material for: Serum IL-1β predicts de novo hepatitis B virus reactivation during direct-acting antiviral therapy for hepatitis C, not during anti-cancer/immunosuppressive therapy
Source: Sci Rep. 2022 Oct 7;12:16800. doi: 10.1038/s41598-022-21315-z (PMC9546937; doi:10.1038/s41598-022-21315-z)
Supplement: Supplementary file 3 — Supplementary Information 3. [file 41598_2022_21315_MOESM3_ESM.docx]

**Supplementary Material**

**Serum IL-1β predicts de-novo hepatitis B virus reactivation during direct-acting antiviral therapy for hepatitis C, not during anti-cancer/immunosuppressive therapy**

Naoki Kawagishi^1¶^, Goki Suda^1¶*^, Ryotaro Sakamori², Takeshi Matsui^3^, Masahiro Onozawa^4^, Zijian Yang¹, Sonoe Yoshida^1^, Masatsugu Ohara^1^, Megumi Kimura¹, Kubo Akinori¹, Osamu Maehara^5^, Qingjie Fu¹, Shunichi Hosoda¹, Yoshimasa Tokuchi¹, Kazuharu Suzuki¹, Masato Nakai¹, Takuya Sho¹, Kenichi Morikawa¹, Mitsuteru Natsuizaka¹, Koji Ogawa^1^, Hajime Sakai^3^, Shunsuke Ohnishi^5^, Masaru Baba^6^, Tetsuo Takehara², Naoya Sakamoto¹^*^

**Table of Contents:**

**Supplementary Table S1**

**Supplementary Table S2**

**Supplementary Table S3**

**Supplementary Figure S1**

**Supplementary Figures and Tables**

Supplementary Table S1. Clinical course and characteristics of HCV-infected patients with HBV reactivation or reappearance during DAA therapy

| No. | 1 | 2 | 3 | 4 | 5 | 6 | 7 | 8 | 9 | 10 | 11 |
| --- | --- | --- | --- | --- | --- | --- | --- | --- | --- | --- | --- |
| Baseline anti-HBs (mIU/mL) | Negative | Negative | Negative | Negative | 10.95 | 11.3 | 13 | Negative | Negative | Negative | Negative |
| DAA treatment duration (weeks) | 12 | 12 | 12 | 12 | 12 | 24 | 24 | 12 | 24 | 12 | 12 |
| Time to HBV reactivation or reappearance after treatment initiation (weeks) | 4 | 4 | 12 | 8 | 12 | 8 | 12 | 4 | 4 | 4 | 4 |
| Time for detecting HBcrAg after treatment initiation (weeks) | N/A | ND | ND | ND | ND | ND | ND | ND | 4 | ND | ND |
| Maximum HBV-DNA level (Log　IU/mL) | 2.7 | 2.4 | <1.3+ | <1.3+ | <1.3+ | 2.0 | <1.3+ | <1.3+ | <1.3+ | <1.3+ | <1.3+ |
| Maximum HBcrAg level (Log U/mL) | N/A | <3.0 | <3.0 | <3.0 | <3.0 | <3.0 | <3.0 | <3.0 | 3.1 | <3.0 | <3.0 |
| Maximum ALT level (IU/L) | 16 | 71 | 80 | 20 | 15 | 18 | 301 | 20 | 41 | 27 | 25 |
| Antiviral therapy for HBV reactivation or reappearance | Not-used | Not-used | Not-used | Not-used | Not-used | Not-used | Not-used | Not-used | Not-used | Not-used | Not-used |

Abbreviations: HCV, hepatitis C virus; HBV, hepatitis B virus; ALT, alanine aminotransferase; anti-HBs, antibody to hepatitis B surface antigen; DAA, direct-acting antiviral, HBcrAg; hepatitis B core-related antigen

Supplementary Table S2. Univariate and multivariate logistic regression analyses of cytokines and clinical factors associated with HBV reactivation or reappearance during DAA treatment

Abbreviations: DAA, direct-acting antiviral; HBV, hepatitis B virus; IL, interleukin; TNF, tumor necrosis factor; anti-HBs, antibody to hepatitis B surface antigen

*Statistically significant difference, P<0.05

|  | **Reactivation** | **Non-reactivation** | **Univariate analysis** | **Multivariate analysis** | **Odds ratio** |
| --- | --- | --- | --- | --- | --- |
| **Number** | **11** | **35** |  |  |  |
| **IL-1α (pg/mL)** |  |  |  |  |  |
| **<10.22, 10.22≤** | **9/2** | **9/26** | ***0.001** | **0.202** |  |
| **IL-1β (pg/mL)** |  |  |  |  |  |
| **<0. 18, 0.18≤** | **9/2** | **3/32** | ***<0.001** | ***<0.001** | **47.6 (6.94–333.3)** |
| **TNFα (pg/mL)** |  |  |  |  |  |
| **<4.813, 4.813≤** | **9/2** | **13/22** | ***0.01** | **0.111** |  |
| **Anti-HBs titer (mIU/mL)** |  |  |  |  |  |
| **Negative or <30, 30≤** | **0/11** | **12/23** | ***0.0021** | **0.282** |  |

Supplementary Table S3. Characteristics of patients with resolved HBV infection receiving anti-cancer/immunosuppressive therapy or bone marrow transplantation

|  | **Reactivation** | **Non-reactivation** | **Univariate analysis** |
| --- | --- | --- | --- |
| **Number** | **6** | **12** |  |
| **Age (years) ^a^** | **64 (55–76)** | **66 (34–78)** | **0.99** |
| **Sex (male/female)** | **5/1** | **5/7** | **0.12** |
| **Primary disease**  **Solid cancer/ blood disorder/** **Autoimmune disease** | **2/2/2** | **0/10/2** | **0.05** |
| **Treatment**  **anti-cancer/immunosuppressive therapy/bone marrow transplantation** | **1/2/3** | **7/2/3** | **0.245** |
| **Platelet count (×10^4^) ^a^** | **13.5 (10.8–27)** | **22.8 (5.6–29.4)** | **0.18** |
| **Albumin (g/dL) ^a^** | **3.8 (3.4–4.1)** | **3.9 (3.1–4.6)** | **0.82** |
| **AST (IU/L)^a^** | **31 (17–72)** | **24 (12–64)** | **0.437** |
| **ALT (IU/L) ^a^** | **23 (11–88)** | **26 (9–81)** | **0.892** |
| **γGTP (IU/L) ^a^** | **30 (12–168)** | **57 (15–559)** | **0.335** |
| **FIB-4 index ^a^** | **2.62 (1.49–7.02)** | **1.57 (0.38–3.75)** | **0.25** |
| **HBV status** |  |  |  |
| **anti-HBs +/-** | **3/3** | **8/4** | **0.428** |
| **Anti-HBs negative or <30 mIU/mL** | **5/1** | **7/5** | **0.306** |

Abbreviations: HBV, hepatitis B virus; AST, aspartate aminotransferase; ALT, alanine aminotransferase; γGTP, γ-glutamyl transpeptidase; anti-HBs, antibody to hepatitis B surface antigen

^a^Data are shown as median (range) values.

*Statistically significant difference, P<0.05

**Supplementary Figure 1.** Comparison of baseline cytokine levels among patients with resolved HBV infection who were treated with anti-cancer/immunosuppressive therapy or bone marrow transplantation, stratified according to the treatment method (anti-cancer/immunosuppressive therapy or bone marrow transplantation) and existence of HBV reactivation during the treatment

Data are shown as median±SD.

* P<0.05, ** P<0.01

BMT, bone marrow transplantation; HCV, hepatitis C virus; HBV, hepatitis B virus; DAA, direct-acting antiviral; IL, interleukin; MCP, monocyte chemotactic protein; IFN, interferon; TNF, tumor necrosis factor; SD, standard deviation; R, HBV reactivation; N-R, non-HBV reactivation; C, HCV control without previous HBV infection; prHBV, previously resolved HBV infection
